# Overnight Monitoring After T&A for Children Ages 24–36 Months: Is It Always Necessary?

**DOI:** 10.1002/lary.70243

**Published:** 2025-11-05

**Authors:** Tyler Van Heest, Luka Bahra, Suhong Tong, Regina Hofner‐Hnotz, Norman Friedman

**Affiliations:** ^1^ Pediatric ENT and Facial Plastic Surgery, Children's Minnesota Minneapolis Minnesota USA; ^2^ Department of Otolaryngology—Head and Neck Surgery University of Minnesota Minneapolis Minnesota USA; ^3^ Department of Otolaryngology—Head and Neck Surgery, Anschutz School of Medicine University of Colorado Aurora Colorado USA; ^4^ Department of Biostatistics and Informatics Colorado School of Public Health Aurora Colorado USA; ^5^ Children's Hospital Colorado Aurora Colorado USA; ^6^ Section of Pediatric Otolaryngology Children's Hospital Colorado Aurora Colorado USA

**Keywords:** child, complications, obstructive sleep apnea, obstructive sleep‐disordered breathing, overnight monitoring, same day surgery, tonsillectomy

## Abstract

**Objectives:**

To determine whether children < 3 years of age who do not require oxygen beyond 3 h and pass an asleep room air challenge (AsRAC), defined as maintaining a SpO_2_ of ≥ 90% for 20 min during sleep, would be safe for discharge after tonsillectomy with or without adenoidectomy (T+/−A).

**Methods:**

All children aged 24–36 months undergoing T+/−A from 2019 to 2021 were included. Demographic, clinical, and polysomnography (PSG) results were stratified based on the presence of a prolonged oxygen requirement (POR) and compared using the Kruskal–Wallis test for continuous variables and Chi‐squared test or Fisher's Exact tests for categorical variables. Univariate and multiple logistic models were performed.

**Results:**

A total of 645 children were included. Overall, 524 (81.2%) successfully weaned from oxygen within 3 h of surgery, and 517 (98.7%) of patients who were off oxygen within 3 h never went back on oxygen during the monitoring period. Patient sex (OR = 1.88 [95% CI, 1.19–2.96]; *p* = 0.006) and diagnosis of chronic lung disease (CLD) (OR = 13.41 [95% CI, 3.69–48.81]; *p* < 0.0001) were the only statistically significant risk factors associated with a POR. No association was found between any of the preoperative PSG variables and a POR.

**Conclusions:**

Children between the ages of 24‐ and 36‐months undergoing T+/−A who have weaned off oxygen within 3 h after surgery and passed an AsRAC would be candidates for same‐day surgery. CLD was the only clinically relevant risk factor for a POR, and no preoperative PSG variables predicted POR.

**Level of Evidence:**

4.

## Graphical abstract

This study aims to identify whether all children < 3 years of age require overnight admission after tonsillectomy with or without adenoidectomy. We found that 98.7% of children who were off oxygen within 3 h after surgery never went back on oxygen during the entire monitoring period. Male gender and chronic lung disease were the only statistically significant risk factors for a prolonged oxygen requirement. In conclusion, children between the ages of 24‐ and 36‐months undergoing T+/−A who have weaned off oxygen within 3 h after surgery and passed an asleep room air challenge would be candidates for same‐day surgery.

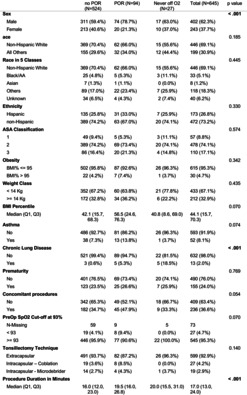

## 1 Introduction

Tonsillectomy with or without adenoidectomy (T+/−A) is one of the most common otolaryngologic procedures, and the most common indication for T+/−A is obstructive sleep disordered breathing (oSDB) [1, 2, 3]. Clinical practice guidelines from both the American Academy of Otolaryngology—Head and Neck Surgery (AAO‐HNS) and the American Academy of Pediatrics (AAP) make recommendations about which children require postoperative monitoring after T+/−A [4, 5]. The AAO‐HNS guideline recommends that all children < 3 years old or with severe OSA (AHI > 10 and/or SpO_2_ nadir < 80%) be observed overnight [4]. The AAP guideline recommends overnight inpatient observation for all patients with an SpO_2_ nadir < 80%, an AHI > 24, or a PCO_2_ > 60 mmHg on preoperative polysomnography (PSG), and that patients with an active upper respiratory illness (URI) should either be rescheduled or monitored postoperatively as well [5].

While the AAP and AAO‐HNS guidelines have been useful for standardizing criteria for overnight observation after T+/−A, there are limitations to these recommendations. For instance, the recommendations about postoperative observation are based on PSG findings, which most pediatric otolaryngologists do not obtain prior to surgery [6, 7]. Additionally, while broad categories based on PSG findings or age are useful for standardizing care, they likely lead to unnecessary admissions that utilize limited healthcare resources and are an inconvenience to families. A pragmatic clinical assessment has the potential to better identify patients at risk for postoperative respiratory events, reduce the number of unnecessary admissions, and provide more individualized care.

In 2021, Friedman et al. published a prospective study showing that patients who are off oxygen within 3 h after extubation and pass a 20‐min asleep room air challenge (AsRAC) are at low risk for postoperative respiratory events and can be discharged home [8]. The study found that there were no postoperative respiratory events for children who were off oxygen within 3 h of extubation among children who were observed overnight. The only statistically significant risk factors associated with a prolonged oxygen requirement (POR) were asthma and Down syndrome. Based on these findings, patients who are off oxygen within 3 h of extubation and pass an AsRAC do not require overnight monitoring, regardless of whether they are considered “high‐risk” by AAO‐HNS/AAP guidelines. While children < 3 years of age were included in the analysis, it did not focus specifically on this cohort and was not sufficiently powered to draw strong conclusions about that subgroup.

The present study aims to determine whether children who are < 3 years of age require overnight monitoring after T+/−A. The authors hypothesize that otherwise healthy children between 24 and 36 months of age who are off oxygen within 3 h and pass an AsRAC do not require overnight monitoring after T+/−A.

## 2 Materials and Methods

Colorado Multiple Institutional Review Board (COMIRB) approval was obtained (COMIRB #19‐2031). Study data were stored in the Research Electronic Data Capture (REDCap) system at the study institution.

**FIGURE 1:**
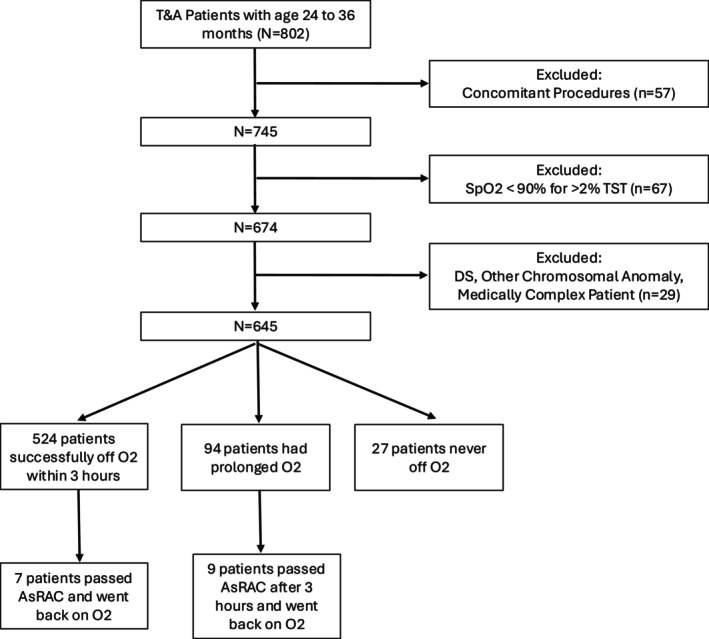
CONSORT flow diagram for study.

Children undergoing a T+/−A between the ages of 24 and 36 months from 2019 to 2021 were included in the study. Patients who had operative airway interventions or non‐otolaryngologic concomitant procedures, complex medical comorbidities (Sickle cell anemia, neuromuscular disorders, Down syndrome, and complex congenital heart disease were the comorbidities excluded) or hypoxemia on preoperative PSG (SpO_2_ < 90% for > 2% of total sleep time) were excluded from the analysis (Figure 1). Preoperatively, patients are given oral acetaminophen, as well as midazolam as needed for anxiolysis (Table 1). Patients undergo inhalational induction and subsequent IV placement. Intraoperative anesthesia is administered with IV propofol and an opioid prior to intubation with an oral RAE tube, and anesthesia is maintained with sevoflurane and propofol. Decadron and ondansetron are administered for anti‐inflammatory and anti‐emetic prophylaxis. Additional opioid and dexmedetomidine are given intraoperatively if clinically indicated. Patients are then extubated after the procedure and brought to PACU. Postoperatively, patients are given oral ibuprofen, as well as fentanyl and/or oxycodone as needed. Patients are transferred to Phase II recovery when alert. Prior to discharge, all patients must pass an AsRAC, tolerate oral intake, and have stable vital signs.

**TABLE 1:** Summary of anesthetic care for tonsillectomy.

Phase of care	Anesthetic plan
Preoperative care	Acetaminophen PO Midazolam PRN for anxiolysis
Intraoperative care	Inhalational induction with subsequent PIV placement IV propofol and opioid prior to intubation with oral RAE IV dexamethasone and ondansetron for anti‐inflammatory and antiemetic prophylaxis Additional opioid and/or dexmedetomidine PRN Extubated and brought to PACU for recovery
Postoperative care	Ibuprofen PO IV fentanyl PRN for severe pain or emergence agitation Oxycodone PRN for patients being discharged with oxycodone prescription Transition to Phase II when modified Aldrete score > 9 or > 8 with anesthesiologist approval Patients must pass AsRAC, tolerate PO intake, and have stable vitals prior to discharge

Demographic information for all participants was collected, including sex, race, and ethnicity. Additional preoperative variables included surgical indication, ASA classification, comorbidities, and preoperative awake SpO_2_. The body mass index percentile (BMI%) for age/sex was calculated per CDC guidelines. PSG data included the obstructive apnea/hypopnea index (oAHI), SpO_2_ nadir, and peak end‐tidal CO_2_. Intraoperative and postoperative variables included procedure duration, postoperative ED visits, and postoperative readmission.

The primary outcome measure was the presence of a POR, which was defined as being on oxygen for greater than 3 h after surgery. A child was considered to have a complication if they were placed back on supplemental oxygen after passing an AsRAC. A successful AsRAC is defined as maintaining a SpO2 of ≥ 90% for a minimum of 20 min. Brief SpO_2_ desaturations to 85% for less than 30 s are allowed. The 3‐h cut off used in the definition of POR was based on the observation that most respiratory events occur within 2 h after surgery and that longer than 3 h of postoperative observation in the recovery area is difficult for same‐day surgery centers to implement. The institutional protocol is to start supplemental oxygen in the recovery area for SpO_2_ persistent < 90%.

Descriptive statistics were summarized for continuous variables with medians and interquartile ranges, and for categorical variables with frequencies and proportions. Group differences are tested via the Kruskal–Wallis test for continuous variables and the *χ* ^2^ test or Fisher's exact tests for categorical variables.

Univariate and multiple logistic models were performed to identify the association between the outcome of interest and potential clinical factors, applying the penalized maximum likelihood method for rare cases. Patient demographics and baseline clinical characteristics with a *p* value less than 0.15 in univariable analyses were selected as covariates for inclusion in the multiple logistic regression models. Patients who had POR and patients who were never off oxygen during the postoperative observation period were combined into one group and compared to the patients who weaned off oxygen within 3 h postoperative as the outcome/dependent variable in the logistic regression model. ROC analysis was used to determine the optimal cut‐offs in predicting POR for the continuous variables in the multiple logistic regression model.

## 3 Results

A total of 802 children between the ages of 24‐ and 36‐months underwent T+/−A during the trial period, and 157 patients were excluded as demonstrated in Figure 1. The remaining 645 children were included in the final analysis. A total of 226 children included in the study had concomitant otolaryngologic procedures (see Table 2). Of the 645 patients included in the analysis, 524 patients successfully weaned from oxygen within 3 h of surgery, 94 patients had a POR, and 27 patients never weaned off oxygen. Overall, 517 of the 524 children (98.7%) who were off oxygen within 3 h never went back on oxygen during the entire monitoring period. Only 7 of 645 (1.1%) patients would have been discharged but were placed back on oxygen in the hospital. Of the seven patients who were placed back on oxygen, five were placed on oxygen following a desaturation to the mid 80s, with no episodes of cardiopulmonary events, reinstating positive pressure, reintubation. It was unclear why the other two children were temporarily placed on oxygen. All these children were discharged the next day on room air. Of the 94 children who were still on oxygen 3 h following surgery, 10 (9.6%) went back on oxygen after they had passed an AsRAC.

**TABLE 2:** Concomitant otolaryngologic procedures performed in included patients.

Concomitant procedures	Number of patients
PET	119
EUA ears	35
MLB	24
EUA ears + other	14
PET + other	8
Frenulectomy	6
MLB + other	5
ABR	4
Nasal endoscopy	2
Inferior turbinate reduction	2
Frenulectomy + other	2
Paper patch myringoplasty	1
Oral lesion excision	1
Flexible laryngoscopy	1
Excision skin lesion	1
DISE	1

Demographic variables are stratified by POR in Table 3. Male sex (*p* < 0.001), diagnosis of chronic lung disease (CLD) (*p* < 0.001), and longer procedure duration (*p* < 0.001) were statistically significant variables for predicting whether a child would have a POR or never come off oxygen. BMI percentile (*p* = 0.070), diagnosis of asthma (*p* = 0.074), and non‐excluded concomitant ENT procedures (*p* = 0.054) were not statistically significant.

**TABLE 3:** Demographic and clinic characteristics stratified by oxygen status at 3 h.

	No POR (*N* = 524)	POR (*N* = 94)	Never off O_2_ (*N* = 27)	Total (*N* = 645)	*p*
*Sex*					**< 0.001**
Male	311 (59.4%)	74 (78.7%)	17 (63.0%)	402 (62.3%)	
Female	213 (40.6%)	20 (21.3%)	10 (37.0%)	243 (37.7%)	
*Race*					0.185
Non‐Hispanic White	369 (70.4%)	62 (66.0%)	15 (55.6%)	446 (69.1%)	
All others	155 (29.6%)	32 (34.0%)	12 (44.4%)	199 (30.9%)	
*Race in five classes*					0.445
Non‐Hispanic White	369 (70.4%)	62 (66.0%)	15 (55.6%)	446 (69.1%)	
Black/AA	25 (4.8%)	5 (5.3%)	3 (11.1%)	33 (5.1%)	
Asian	7 (1.3%)	1 (1.1%)	0 (0.0%)	8 (1.2%)	
Others	89 (17.0%)	22 (23.4%)	7 (25.9%)	118 (18.3%)	
Unknown	34 (6.5%)	4 (4.3%)	2 (7.4%)	40 (6.2%)	
*Ethnicity*					0.330
Hispanic	135 (25.8%)	31 (33.0%)	7 (25.9%)	173 (26.8%)	
Non‐Hispanic	389 (74.2%)	63 (67.0%)	20 (74.1%)	472 (73.2%)	
*ASA classification*					0.574
1	49 (9.4%)	5 (5.3%)	3 (11.1%)	57 (8.8%)	
2	389 (74.2%)	69 (73.4%)	20 (74.1%)	478 (74.1%)	
3	86 (16.4%)	20 (21.3%)	4 (14.8%)	110 (17.1%)	
*Obesity*					0.342
BMI% ≤ 95	502 (95.8%)	87 (92.6%)	26 (96.3%)	615 (95.3%)	
BMI% > 95	22 (4.2%)	7 (7.4%)	1 (3.7%)	30 (4.7%)	
*Weight class*					0.435
< 14 kg	352 (67.2%)	60 (63.8%)	21 (77.8%)	433 (67.1%)	
≥ 14 kg	172 (32.8%)	34 (36.2%)	6 (22.2%)	212 (32.9%)	
*BMI percentile*					0.070
Median (Q1, Q3)	42.1 (15.7, 68.3)	56.5 (24.6, 76.3)	40.8 (8.6, 69.0)	44.1 (15.7, 70.3)	
*Asthma*					0.074
No	486 (92.7%)	81 (86.2%)	26 (96.3%)	593 (91.9%)	
Yes	38 (7.3%)	13 (13.8%)	1 (3.7%)	52 (8.1%)	
*Chronic lung disease*					**< 0.001**
No	521 (99.4%)	89 (94.7%)	22 (81.5%)	632 (98.0%)	
Yes	3 (0.6%)	5 (5.3%)	5 (18.5%)	13 (2.0%)	
*Prematurity*					0.769
No	401 (76.5%)	69 (73.4%)	20 (74.1%)	490 (76.0%)	
Yes	123 (23.5%)	25 (26.6%)	7 (25.9%)	155 (24.0%)	
*Concomitant procedures*					0.054
No	342 (65.3%)	49 (52.1%)	18 (66.7%)	409 (63.4%)	
Yes	182 (34.7%)	45 (47.9%)	9 (33.3%)	236 (36.6%)	
PreOp SpO_2_ cut‐off at 93%					0.070
*N* missing	59	9	5	73	
< 93	19 (4.1%)	8 (9.4%)	0 (0.0%)	27 (4.7%)	
≥ 93	446 (95.9%)	77 (90.6%)	22 (100.0%)	545 (95.3%)	
*Tonsillectomy technique*					0.140
Extracapsular	491 (93.7%)	82 (87.2%)	26 (96.3%)	599 (92.9%)	
Intracapsular—coblation	19 (3.6%)	8 (8.5%)	0 (0.0%)	27 (4.2%)	
Intracapsular—microdebrider	14 (2.7%)	4 (4.3%)	1 (3.7%)	19 (2.9%)	
*Procedure duration (min)*					**< 0.001**
Median (Q1, Q3)	16.0 (12.0, 23.0)	19.5 (16.0, 26.8)	20.0 (15.5, 31.0)	17.0 (13.0, 24.0)	

*Note*: Bold values indicate statistically significant *p *< 0.05.

A univariate logistic regression model was performed (Table 4). Male sex (OR = 2.08 [95% CI, 1.33–3.25]; *p* = 0.001), diagnosis of CLD (OR = 14.0 [95% CI, 3.93–50.1]), and non‐excluded concomitant ENT procedures (OR = 1.52 [95% CI, 1.01–2.26]; *p* = 0.042) were all found to be significant predictors of POR.

**TABLE 4:** Univariate logistic regression model.

Odds ratio estimates				
Effect	Odds ratio	95% Wald		
		Confidence limits		*p*
Sex, M vs. F	2.08	1.33	3.25	**0.001**
Asthma, Y vs. N	1.67	0.88	3.2	0.119
CLD, Y vs. N	14.03	3.93	50.06	**< 0.001**
Concomitant procedures Y vs. N	1.52	1.01	2.26	**0.042**
MLB Y vs. N	2	0.95	4.18	0.067
PreOp_Spo_2__C93, < 93 vs. ≥ 93	1.9	0.81	4.46	0.142
BMIPCT	1.01	1	1.01	0.086
Procedure duration (min)	1.01	1	1.03	0.057

*Note*: Bold values indicate statistically significant *p* < 0.05.

A multiple logistic regression model was then performed (Table 5). After adjusting for all potential factors in the model, patient sex and diagnosis of CLD were the only statistically significant risk factors associated with a POR. Male patients had 1.88 times higher odds of having a POR compared to female patients. Patients with CLD had 13.4 times higher odds of having a POR compared to patients without CLD. BMI percentile was marginally significant (OR = 1.01 [95% CI, 1.00–1.01]; *p* = 0.053).

**TABLE 5:** Multiple logistic regression model.

Odds ratio estimates				
Effect	Odds ratio	95% Wald		
		Confidence limits		*p*
Sex, M vs F	1.88	1.19	2.96	**0.006**
CLD, Y vs N	13.41	3.69	48.81	**< 0.001**
BMIPCT	1.01	1.00	1.01	0.053

*Note*: Bold values indicate statistically significant *p* < 0.05.

A total of 107 children included in the study underwent preoperative PSG (Table 6). Six (5.6%) patients had no OSA, 36 (33.6%) patients had mild OSA, 38 (35.5%) had moderate OSA, and 27 (25.2%) had severe OSA. Of the patients with severe OSA, five (18.5%) had an AHI > 20. Thirty (28.0%) patients with preoperative PSG had SpO_2_ nadir < 80%. Twelve (11.4%) patients who underwent preoperative PSG had a peak end tidal CO_2_ of > 50. Both univariate and multiple logistic regression models were performed, and no association was found between any of the preoperative PSG variables and a POR.

**TABLE 6:** Polysomnography data stratified by prolonged oxygen requirement.

	No POR (*N* = 91)	POR (*N* = 9)	Never off O_2_ (*N* = 4)	Off O_2_ back O_2_ (*N* = 3)	Total (*N* = 107)
*OSA severity index*					
< 1	5 (5.5%)	1 (11.1%)	0 (0.0%)	0 (0.0%)	6 (5.6%)
1 to < 5	32 (35.2%)	1 (11.1%)	1 (25.0%)	2 (66.7%)	36 (33.6%)
5 to < 10	31 (34.1%)	5 (55.6%)	2 (50.0%)	0 (0.0%)	38 (35.5%)
10 to < 20	18 (19.8%)	2 (22.2%)	1 (25.0%)	1 (33.3%)	22 (20.6%)
≥ 20	5 (5.5%)	0 (0.0%)	0 (0.0%)	0 (0.0%)	5 (4.7%)
*SpO*_*2*_ *nadir*					
≥ 80%	66 (72.5%)	5 (55.6%)	3 (75.0%)	3 (100.0%)	77 (72.0%)
< 80%	25 (27.5%)	4 (44.4%)	1 (25.0%)	0 (0.0%)	30 (28.0%)
*Peak end tidal CO*_*2*_					
*N*—missing	1	1	0	0	2
≤ 50	79 (87.8%)	7 (87.5%)	4 (100.0%)	3 (100.0%)	93 (88.6%)
> 50	11 (12.2%)	1 (12.5%)	0 (0.0%)	0 (0.0%)	12 (11.4%)

A total of 599 (92.9%) patients underwent extracapsular tonsillectomy, and 36 (7.1%) patients underwent intracapsular tonsillectomy. There was no significant difference in risk for a POR either by *χ* ^2^ test (*p* = 0.142).

## 4 Discussion

The AAO‐HNS CPG for tonsillectomy recommends that all children less than 3 years of age regardless of their OSA severity, gas exchange, BMI% or being healthy should be monitored overnight [4]. A number of pediatric otolaryngology groups are performing same‐day surgery for toddlers [6]. The current investigation corroborates our previous research that there is no association of age with a POR [8]. Over 80% of children between the ages of 24 and 36 months were weaned off oxygen within 3 h after surgery and only 1% subsequently went back on oxygen after passing an AsRAC. All children who were started back on oxygen were discharged home the following morning on room air. These findings suggest that a child who has successfully weaned off oxygen within 3 h is likely safe to discharge home from a respiratory standpoint.

Our study also demonstrated that a POR after T+/−A relates more to underlying lung health than postoperative upper airway obstruction. The multiple logistic regression model found that a diagnosis of CLD was associated with a POR. The clinical significance of this association is high with an odds ratio > 13. A multiple logistic regression model of the preoperative PSG data found no significant association between OSA severity, SpO_2_ nadir, or peak end tidal CO_2_ and requiring oxygen for > 3 h. These findings taken together suggest that underlying pulmonary disease is what puts patients at increased risk for a POR after anesthesia, rather than postoperative upper airway obstruction from oSDB. Although an asthma diagnosis was not a statistically significant predictor of a POR, it is prudent that asthma is under control prior to surgery and that the child does not have an upper respiratory infection [9].

Overnight observation of children < 3 years old after T+/−A is common but not a universal practice. A survey of all members of the American Society of Pediatric Otolaryngology regarding their postoperative admission practices after T+/−A found that 80% of respondents admitted all patients < 3 years old after tonsillectomy [10]. Similarly, most pediatric otolaryngology division chiefs at pediatric tertiary care hospitals identify age < 3 years old as an indication for overnight monitoring after T+/−A at their institution [11].

However, the evidence that children < 3 years old are at higher risk for perioperative morbidity is equivocal. Biadsee et al. published a prospective case series comparing postoperative complication rates in 92 children who underwent intracapsular tonsillectomy for SDB, and found no difference in complication rates between patients < 3 years old and older children (5.7% vs. 8.8%; *p* = 0.59) [12]. Similarly, Belyea et al. in 2014 performed a retrospective case–control study comparing postoperative complication rates after T+/−A between 127 children < 3 years old with 127 matched controls who were 3 years old [13]. The authors found no statistically significant difference in postoperative complication rates (9.4% vs. 8.7%; *p* > 0.05). While both studies are limited by a lack of statistical power, they do suggest that routine overnight monitoring of all children < 3 years old may be unnecessary.

The use of a pragmatic approach to identifying children at risk for postoperative complications may be more effective than admitting based on age alone. Previous research by the senior author has shown that an AsRAC effectively identifies children who have low risk for significant postoperative respiratory events after T+/−A [8]. Subsequent research has shown that using an AsRAC in the recovery area to identify and discharge low risk patients increases the rate of same day surgeries (58% vs. 74%; *p* < 0.05) with no difference in ED revisit rate or postoperative respiratory events [14].

The current study supports our hypothesis that children between the ages of 24‐ and 36‐months undergoing T+/−A who have weaned off oxygen within 3 h after surgery are unlikely to have a perioperative respiratory adverse event or develop an oxygen requirement once they pass an AsRAC. An AsRAC is critical since sleep will unmask respiratory insufficiency and be a warning sign that a longer observation period is warranted. Using this approach, over 80% of eligible children could have had same‐day surgery, and all children with a POR would have still been observed overnight. Rather than mandatory overnight observation, we are advocating for a paradigm shift where otherwise healthy toddlers including those who have an elevated oAHI have surgery at a facility that could perform overnight monitoring if needed. While there is insufficient evidence currently to change the AAO‐HNS guidelines, we do think there is promise with this new paradigm shift for reducing unnecessary admissions.

Strengths of the study include the size of the case series. The study also includes children who underwent concomitant procedures as well as both intracapsular and extracapsular tonsillectomy techniques, improving the external validity compared to studies that look at one technique or the other. The study also includes preoperative PSG data when it is available.

Limitations of the study include the retrospective study design, reliance on nursing and other healthcare staff to administer and document supplemental oxygen accurately, reliance on staff at an institution where performing AsRAC is routine, and no control group to compare children < 3 years old to older children.

We have modified our current overnight monitoring criteria allowing children over 2 years of age to have same‐day surgery if they pass an AsRAC and are currently tracking unanticipated ED visits or readmissions to help verify the safety of this approach.

## 5 Conclusion

In conclusion, the current study demonstrates that children between the ages of 24‐ and 36‐months undergoing T+/−A who have weaned off oxygen within 3 h after surgery and passed an AsRAC rarely are placed back on oxygen. CLD was the only clinically relevant risk factor for a POR, and no preoperative PSG variables predicted POR. Many 2‐year‐olds are candidates for same‐day surgery. Additional investigations are ongoing to confirm our findings. T+/‐As on younger children should still be performed at facilities where there is an option to monitor them overnight if necessary.

## Conflicts of Interest

The authors declare no conflicts of interest.

## Data Availability

The data that support the findings of this study are available on request from the corresponding author. The data are not publicly available due to privacy or ethical restrictions.
